# Recruiting Terminally Ill Patients into Non-Therapeutic Oncology Studies: views of Health Professionals

**DOI:** 10.1186/1472-6939-13-33

**Published:** 2012-12-05

**Authors:** Erika Kleiderman, Denise Avard, Lee Black, Zuanel Diaz, Caroline Rousseau, Bartha Maria Knoppers

**Affiliations:** 1 Centre of Genomics and Policy, Faculty of Medicine, Dept of Human Genetics, McGill University, 740 Dr. Penfield Avenue, 5th Floor, Suite 5200, Montreal, (QC) H3A 1A4, Canada; 2 Québec-Clinical Research Organization in Cancer (Q-CROC), Jewish General Hospital, 3755 Côte Ste-Catherine Road, Suite E-445, Montreal, (QC), H3T 1E2, Canada

**Keywords:** Non-therapeutic, Health professional, Bioethics, Consent, Interview, Terminally ill

## Abstract

**Background:**

Non-therapeutic trials in which terminally ill cancer patients are asked to undergo procedures such as biopsies or venipunctures for research purposes, have become increasingly important to learn more about how cancer cells work and to realize the full potential of clinical research. Considering that implementing non-therapeutic studies is not likely to result in direct benefits for the patient, some authors are concerned that involving patients in such research may be exploitive of vulnerable patients and should not occur at all, or should be greatly restricted, while some proponents doubt whether such restrictions are appropriate. Our objective was to explore clinician-researcher attitudes and concerns when recruiting patients who are in advanced stages of cancer into non-therapeutic research.

**Methods:**

We conducted a qualitative exploratory study by carrying out open-ended interviews with health professionals, including physicians, research nurses, and study coordinators. Interviews were audio-recorded and transcribed. Analysis was carried out using grounded theory.

**Results:**

The analysis of the interviews unveiled three prominent themes: 1) ethical considerations; 2) patient-centered issues; 3) health professional issues. Respondents identified ethical issues surrounding autonomy, respect for persons, beneficence, non-maleficence, discrimination, and confidentiality; bringing to light that patients contribute to science because of a sense of altruism and that they want reassurance before consenting. Several patient-centered and health professional issues are having an impact on the recruitment of patients for non-therapeutic research. Facilitators were most commonly associated with patient-centered issues enhancing communication, whereas barriers in non-therapeutic research were most often professionally based, including the doctor-patient relationship, time constraints, and a lack of education and training in research.

**Conclusions:**

This paper aims to contribute to debates on the overall challenges of recruiting patients to non-therapeutic research. This exploratory study identified general awareness of key ethical issues, as well as key facilitators and barriers to the recruitment of patients to non-therapeutic studies. Due to the important role played by clinicians and clinician-researchers in the recruitment of patients, it is essential to facilitate a greater understanding of the challenges faced; to promote effective communication; and to encourage educational research training programs.

## Background

Non-therapeutic trials in which terminally ill cancer patients are asked to undergo procedures such as biopsies or venipunctures for research purposes, have become increasingly important to learn more about how cancer cells work and to realize the full potential of clinical research. Considering the increased use of specimen collection in clinical trials within the context of emerging ‘personalized’ medicine, one ethical issue looming over the research community concerns whether cancer patients should be recruited to non-therapeutic oncology research [1]. These studies may involve patients who are in advanced stages of their disease, whose life expectancies are limited [2], and for whom research raises distinct ethical, as well as logistical challenges. For this type of research the use of oncology patients is necessary, because healthy participants cannot offer access to tumor cells.

Considering that implementing non-therapeutic studies is not likely to result in direct benefits for the patient, some authors are concerned that involving patients in such research may be exploitive of vulnerable patients and should not occur at all, or should be greatly restricted [3]. Alternatively, some proponents doubt whether such restrictions are appropriate [3]. These proponents view the terminally ill as autonomous individuals, able to engage in decision-making; thus any restrictions to this kind of research are seen as paternalistic and a form of devaluing their personal autonomy and self-determination [4]. Although research ethics boards (REBs) are essential for the general oversight of human subject research, this ambivalence about whether the risks of exploitation or harm are important enough to limit such research has been a source of debate at the ethics review committee level [3]. Discussions about recruiting patients to non-therapeutic research have been questioned for a variety of ethical reasons, including: i) increased vulnerability; ii) reconciling researcher and clinician roles; and iii) familial considerations.

### Increased vulnerability

Patients in advanced stages of cancer, who are within days, weeks or months of dying are the target population for non-therapeutic oncology studies. They often experience severe physical and psychological problems [5]. These patients are considered highly vulnerable because they are very ill, have decreased cognitive abilities, have severe pain, nausea, anxiety, or have difficulty accepting their approaching death. Moreover, their physiological state can change rapidly, such that one day they are fine and the next day they are preoccupied by something else. As a result, patients’ motivation to participate may fluctuate. For example, at times, they may be motivated to participate for altruistic reasons and for the benefit of other cancer patients, while at other times, they may wish to spend their restricted and valuable time with their families [3].

### Reconciling researcher and clinician roles

Clinician-researchers who provide patient care find it challenging to balance their clinical and researcher roles [6]. For example, the need to act in the patient’s best interest (beneficence) and the risk of infection, causing pain, or other complications as a result of obtaining a biopsy for research raises concerns. Moreover, since human biological samples are important sources of DNA, this may also raise concerns about confidentiality and insurance discrimination [7-9] or give rise to a unique set of medical-ethical dilemmas about informing other family members of pertinent findings [10,11].

The clinician-researcher may also be confounded by issues arising from the therapeutic misconception. Despite clear information on the lack of personal benefit, patients may still feel that the research could offer a promising treatment. They may have unrealistic expectations or see it as an individualized treatment plan recommended by their doctor [12].

In addition, clinicians may have ethical conflicts stemming from the limited understanding of their role in research. They may also be reluctant to approach their patients about the prospect of participation because of their relationship with them, potentially competing loyalties, and a wish to consider the wellbeing of the patient first [6].

### Familial considerations

Families often believe terminally ill patients are less likely to want to participate or to tolerate additional stress or pain [13,14]. There may be times where the family will object or become over-protective.

Effective communication between physicians and patients and their families is essential to high quality cancer care and constitutes one of the greatest challenges faced by physicians [15,16]. It requires physicians to become sensitive to their patient’s emotions and to demonstrate patience, understanding, empathy, and support [16,17]. Patients’ needs vary throughout the course of their disease and with that, so does their need to be kept informed [18]. As a result, different approaches need to be taken to adapt and to help them cope [18,19].

In short, how health professionals approach patients for participation in non-therapeutic research is a sensitive and important issue. It requires a better understanding of the issues to ensure that both science and the patient’s needs are served. While there are numerous ethical and social issues surrounding participation in research at the end of life, there is less of an understanding of how clinicians and researchers address these challenges. This information is important in that clinicians may play a key role in informing their cancer patients about this type of research while being sensitive to patient needs and wishes.

Acknowledging the complexity of the issue, we conducted a qualitative exploratory study to assess clinician-researchers’ attitudes when recruiting patients in advanced stages of cancer into non-therapeutic research. Non-therapeutic research is defined as research not having “direct benefits for participants but [that] may have future benefits for others” [20]. Advanced stages of cancer refers to those who are in Stage III (“Higher numbers indicate more extensive disease: Larger tumor size and/or spread of the cancer beyond the organ in which it first developed to nearby lymph nodes and/or organs adjacent to the location of the primary tumor”) or Stage IV (“The cancer has spread to another organ(s)”) of their cancer [21].

For the purpose of this paper, the term “respondents” refers to the seven interviewees (i.e. participants in this qualitative study), whereas the term “patients” refers to those individuals who would take part in non-therapeutic oncology research (i.e. terminally ill individuals with cancer).

## Methods

### Study participants

Respondents were recruited to ensure a variability of experiences (e.g. gender, age, speciality) and included a total of seven health professionals: three physicians, two research nurses, and two study coordinators, all of whom are involved in oncology research. We used a combination of purposeful and convenience sampling by asking the clinical research team and respondents to suggest other individuals (snowballing), by advertising on the GEOQ (Groupe d’étude en oncologie du Québec) website, and through direct contact with researchers of the Q-CROC Consortium (Québec – Clinical Research Organization in Cancer), a consortium bringing together researchers and clinicians throughout Quebec, including hospitals and research centers. We chose a qualitative method because it allows for exploring processes that lead to a better understanding of the different reasons for including or not including patients in non-therapeutic research. Ethics approval was obtained from the McGill University Institutional Review Board.

### Data collection

Information was collected between 2010 and 2011 using open-ended interviews at the time and location of the respondent’s choice. The interview method was chosen because it is more flexible and adjustable to the availability of the health professionals. The goal was to gain insight into the perceptions of healthcare providers regarding patient recruitment and participation in non-therapeutic research. This study was limited to exploring the issues involving the recruitment of an adult population; thus excluding terminally ill minors. The interview questions and guide were developed on the basis of a review of the literature.

### Sample Interview questions

Attitudes and knowledge of research

What do you think about having your patient participate in a research protocol?

What do you think the physician’s role is? The researcher’s role?

Have you ever referred patients to a research project?

Have you ever declined referral of patients to a research project?

Barriers and facilitators to involving patients in research

What do you think about research that has no obvious therapeutic benefit?

Should any patient be asked to participate in research without therapeutic benefit?

Are there barriers that may contribute to your decision to refer a patient or not?

Are there benefits to participating in research with no obvious therapeutic benefits?

Who should be involved in the consent? How should consent be obtained?

What types of information do you or would you provide to patients?

Should all physicians be asked to participate in research recruitment?

What factors lead to encouraging or discouraging patient participation?

Tools for integrating research practice

What tools could you use to address the introduction of research protocols in your clinic? (Probes: policy statement, incentives, educational, etc.)

Pilot testing of the questionnaire was carried out by the multidisciplinary research team. Interviews lasted 45–60 minutes and were performed by an experienced researcher (DA), in the company of another researcher (LB).

### Data analysis

Interviews were audio-recorded and transcribed, and responses to open-ended questions were coded by two individuals (EK and DA). They were analyzed using a constant comparative method, which inductively seeks to identify themes that emerge across the interviews [22]. A predetermined set of codes was developed from the interview guide and the relevant literature; however, new codes were also allowed to emerge from the data.

**Figure 1:**
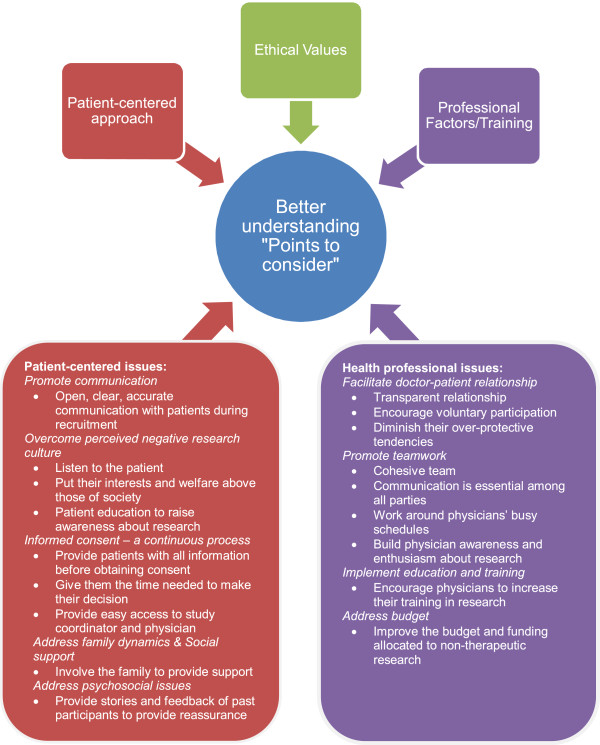
“Points to consider” to facilitate understanding.

The data were iteratively coded using NVivo, a software for qualitative research and analysis (EK). Then, a second individual (DA) also coded a subset of the data for themes. Information was categorized according to themes. We then took the many codes and through consensus merged them into a handful of themes. Each was subsequently classified by an overall enhance or deter factor. The findings are presented with quotes to illustrate the themes that emerged. A model was developed to describe the relationship between the key themes and a range of points to consider (see Figure 1).

## Results

Our analysis of the data reveals three prominent themes: 1) ethical considerations; 2) patient-centered issues; and 3) health professional issues. The results are presented under these three themes. Table 1 contains an overview of the issues.

**Table 1:** Prominent themes

**I. Ethical considerations with respect to non-therapeutic research**	
· Autonomy	
· Respect for persons	
· Beneficence	
· Non-maleficence	
· Discrimination (e.g. insurance and employment)	
· Confidentiality	
· Public good	
**Patient-centered issues**	
**Facilitators**	**Barriers**
· Communication	· Negative research culture
· Informed consent is a continuous process	· Vulnerability of patient
	· Differing opinions
· Research nurse	· Psychosocial issues
· Individual contact	
· Timing	
· Family dynamics and social support	
**II. Health professional issues**	
**Facilitators**	**Barriers**
· Doctor-patient relationship	· Doctor-patient relationship
· Teamwork	· Distinction between care and research
	· Time constraints
	· Conflict of interest
	· Lack of awareness
	· Education and training
	· Research ethics boards (REB)
	· Funds

### I) Ethical considerations

There was consensus that certain core ethical principles should be observed when interacting with patients and their families. When invited to describe their understanding of ethics in the context of non-therapeutic research most respondents articulated the need for:

### Autonomy

A primary ethical principle is that participation in any kind of research requires a free choice (voluntariness) and appropriate information about the research as well as the time to better understand the information being presented to them before consenting. To that end, a potential research participant must be eligible and capable of providing a voluntary and informed consent.

“[…] the patient will never sign the first [day], even though we explain it to them, they will say I’m going to sign now. We say you go home, you read it properly and you come back to see me next week. That’s the way we proceed, because we want them to collaborate, we want them to understand […]” (Interviewee 3)

### Respect for persons

Respondents recognize that because the patients have suffered a lot, and perhaps want to be left alone, their interests and welfare must always take precedence over the interests of science and society. As a matter of respect for human dignity and to avoid treating people as a means to an end, physicians must consider the patient as a human being and demonstrate sensitivity and empathy to his or her needs and emotions.

Many respondents stated that given the sensitive nature of the recruitment, it is also important to listen to the patient and to understand his or her personal situation, because they noted the physician has an ethical and legal duty to promote the best interests of the patient while knowing that the investigation can be stressful, particularly if there are no immediate benefits. In such a scenario, it is appropriate to take into consideration the best interest of the individual.

“[…] we have to choose, we have to really be rational and look at […] the patient as [a] human being, not as […a] case number – it’s a human being.” (Interviewee 5)

“Physicians really feel that […in their] relationship […with their patient,] they have to do what’s best for their patient and it’s their judgment that has to decide that.” (Interviewee 7)

### Beneficence

When asked to describe the positive aspects of this type of research, some respondents suggested that as a result of participating in research, patients may receive better care because it provides them with the opportunity to be treated by specialists and to be closely monitored. In fact, as the following text expresses, if they participate in research, they may benefit because:

“There’s just more hands involved and the scheduling is fixed. So a scan is done. It’s done according to when it should be done. And so […] I think that’s a fringe benefit. I think there are all these psycho-spiritual benefits that are real and true.” (Interviewee 1)

### Non-maleficence

With non-therapeutic research involving biopsies, for example, many respondents highlighted the potential to cause distress for the participant, especially because there is no anticipated physical benefit and greater than minimal risk. When assessing risk, respondents felt it was important to address the risks that may be significant (i.e. increased pain or side effects), prior to participation in research.

“they were already worried about the procedure […] because they didn’t want their father to have pain or discomfort or […] risk[s] simply for […] research purposes.” (Interviewee 2)

Moreover, some recognized that patients are emotionally drained and overwhelmed. As a result, there is an ethical obligation to be cautious about causing distress and to make them feel like partners during the recruitment process.

“I think people are in distress and feel overwhelmed and […] it’s for us to say, okay, look, let’s talk about this tomorrow. Today, you […] digest what you’re facing. I wish you didn’t have to face this but you do. And we’ll talk about the implications tomorrow. It’s a process”. (Interviewee 1)

Furthermore, some health professionals recognized that genetic information that may arise from genomic research may be a source of discrimination. Examples of discrimination included denial of insurability and employability, for the patient as well as for his or her family.

“Something that can allow a family to be targeted and identified then I think this would […be] an element that would […] turn away people from the study because they don’t want the stigma for themselves but also they don’t want a stigma for their families.” (Interviewee 4)

“[insurability] or employability. Those are elements that you know eventually might be detrimental for the whole […] quality of life […] of not only the patient but the others around them.” (Interviewee 4)

### Public good

Many respondents mentioned that the benefits of non-therapeutic research include better knowledge and more insight into the disease rather than individual benefits for the patients. They also mentioned that participation in non-therapeutic research was an altruistic gesture that contributes to the greater good. According to respondents, participation is a way of directly helping their families to avoid a similar situation and to improve the health of future generations.

“For cancer patients that have more advanced [stages of a] disease, there […are] other elements that are important to the patient that ha[ve] to do with their mortality and contributing. There’s some kind of altruism that evolves, contributing to the greater good and to the benefit of others.” (Interviewee 1)

Some also stated that patients are sometimes more than happy to encourage researchers to use these biological samples to help someone else and for future medical advancements.

### II) Patient-centered issues

Several patient-centered issues have an impact on the recruitment of patients for non-therapeutic research. Some issues facilitate the process and others are described as barriers.

### Communication

Fostering open, clear and accurate communication with patients during the recruitment process is seen as essential. Specifically, communication can facilitate recruitment by ensuring that the patient will understand every step of the research process, that their questions will be addressed, and that they will receive sufficient support.

### Negative research culture

Many respondents mentioned that there is much erroneous information about clinical trials in the public domain. Some highlighted the concern that patients may perceive themselves as guinea pigs when participating in non-therapeutic research, perhaps as a result of misinformation over the years.

“I’d say five years ago, a very strong sense of […] guinea pigs [existed], and there was a lot of misinformation about clinical trials.” (Interviewee 1)

A compelling counter argument is to ensure that the patients receive reliable information and understand the real goal of the research. Another approach is to host conferences and discussion groups with patients and participants to enhance the exchange of experiences and information about research and the research sites. One effect may be to reduce the fear of being a guinea pig, for example:

“[a] whole day on clinical research [was set up] last year […]. [T]he theme [was the idea of being a guinea pig in research] and we […] had patients […] and researchers [discuss on the matter]. […] we put a lot of literature out in the waiting room, which stimulate[d] conversation […and] the patients [had the opportunity to] exchange a lot [among] themselves. Are you on this study? Are you on that study? And so, […it encouraged] a culture of research.” (Interviewee 1)

### Informed consent is a continuous process

This is especially true in non-therapeutic research where the primary goal is not to provide clinically relevant results that can benefit the individual. Many respondents viewed the process of informed consent as an interactive flow of ideas. There were indications regarding what information to provide, how to inform the patient, and when to obtain his or her consent.

When obtaining permission from patients, respondents highlighted the need for a thorough explanation of the research process, fully disclosing what will happen.

“I explain everything from A to Z [to] the patient. And then I introduce the study coordinator who then goes through the, kind of walks it through on a, the logistics of it. Like what each day will happen. Then we give them a consent form. And I tell them they must ask me two or three questions at least.” (Interviewee 1)

Another respondent suggested avoiding a group setting for recruitment in favour of individual contacts. This approach sets the stage for individual autonomy by adequately answering patients’ questions and providing easy access to the study coordinator and/or the physician, which are important steps for continuous support.

Timing of the consent process is an equally important issue. Decisions about accepting to participate should not occur on the first day or immediately after receiving all the information. Patients are encouraged to take the information home with them, so that they can think it through before making their decision to consent. Asking questions is also strongly encouraged by the health professionals, who should make themselves available for questions, to ensure that the patient fully comprehends the research and has no hesitations.

Mention was made of the importance of informational tools for patients to guide the consent process. When multiple languages are spoken in a particular population (French and English in Quebec), such tools should be available in these languages to promote and encourage participation in research.

### Family dynamics and social support

Respondents identified the need for patients to feel supported by their families, because of the varying emotional needs and the anxiety associated with treatment, especially when there is little hope for survival.

“The family is always important, […] to be there for the treatment because the treatment is very hard, it’s very tough and they [really] need […] support because even if there is hope, the patient will go up and down with depression, with a lot of things which are really expected and they need support, but when it’s hopeless, they really need to […have parents and family around as…companions…]” (Interviewee 5)

An issue that may arise in the familial context is entanglement, meaning differing opinions between patients and their families regarding participation in non-therapeutic research. Respondents noted that families sometimes adopt a protective approach. As a result, there are times when involving the family is challenging due to these differences in opinions and views, and when patients may fear burdening their families at a sensitive time. For example:

“He agreed to enter the study but you know, most of the time, in this case and in all the other case[s], the patient doesn’t want to do something that can give more concern to his family.” (Interviewee 2)

Interestingly the outlook and opinions of the patient with an advanced illness may also lead to a change in priorities, and as a result influence willingness to participate. Therefore, conferring with families during the process is important, as they can provide a support system for the patient throughout the duration of the study.

### Psychosocial issues

The lowered expectation of survival faced by these patients can create considerable psychological stress. Respondents noted that the patients are emotionally labile at the time of recruitment. These include: ups and downs, anxiety, fear, overwhelmed (they need down time or time to let their emotions subside), and reassurance. Health professionals can make important contributions by supporting and sharing information and insights about the research with patients. Specifically, sitting with the patient and the family to discuss the research protocol is likely to provide a better awareness of the difficulties and hopes of the patients and their families.

“It’s […a] totally different approach. Here we should sit with the patient and with the family and we should really be very comprehensive because [he] is hopeless and […] has a lot of things to face, he is facing a very bad disease, he is going through treatments and he doesn’t have hope so he is facing death and this is really something big to deal with, and it depends on the patient.” (Interviewee 5)

Respondents suggested that another positive communication resource that facilitates a more positive experience is to provide patients with true stories from other patients. By listening to other patients, this reassures them that they are not the first ones to participate in such research.

### III. Health professional issues

A final theme emerging from the interviews are the facilitators and barriers with respect to members of the health professional team (Table 1). Many respondents expressed concerns about the difficulties of carrying out non-therapeutic research.

### Doctor-patient relationship

In order to foster a feeling of trust, respondents highlighted the importance of the doctor-patient relationship. This relationship should be a transparent one, where communication is valued and encouraged.

“You have to be transparent. […] so [the patients] know that our goal is to make everybody comfortable and to give support in all of this.” (Interviewee 3)

Moreover, the physician who cares for the patient may also play the role of gate keeper to research participation or may add pressure on the patient. For example, a physician may feel that a patient has suffered enough and for this reason denies researchers access to eligible participants. In order to protect the patient, physicians may perceive him or her as being unwilling to participate and make this decision for him or her.

“if this patient is not appropriate in a sense that she suffered a lot, […] leave her alone, I’m pretty sure that I will have another case for my research […]” (Interviewee 5)

As a result, physicians sometimes partake in recruitment for a research project only if they believe in its potential to be beneficial. In some cases, the physician may subconsciously communicate disinterest and hesitations to the patient, thereby setting different priorities in the recruitment process.

“I think that we should not force physicians to participate in the project; they have to believe in those projects.” (Interviewee 6)

### Distinction between care and research

In some cases, the issue is that patients may think that participation in research may have a therapeutic value or provide another chance for survival. This is particularly difficult when the patient has trust and confidence in the physician. On the one hand, the patient may feel reassured that the physician is involved, which may encourage participation. On the other hand, it may result in the patient participating because he or she thinks that since the physician suggested it, it must be appropriate (the patient is being influenced by the association made between the physician and good, so it is not necessarily a voluntary decision, but one that has been indirectly influenced by the physician – *therapeutic misconception*).

“Usually that’s the way it is, but they don’t have any idea about what the research is and you know that when a physician [proposes something], it’s really different from a nurse talking about research because usually the patients have confidence in their physician so they say, okay, if my oncologist is proposing the protocol, it means that this is good for me.” (Interviewee 5)

### Teamwork

Cohesive study teams and teamwork between physicians, researchers and the research nurses were perceived as facilitators when conducting non-therapeutic research. Communication should be transparent and a lack of communication makes it more difficult and stressful on the patient and can cause anxiety.

“The physicians […] evaluate the protocol first and then they discuss […] it in a team meeting and after that […] they kind of select the protocols that they want to implement on […their] side or don’t want to implement and only after that will we start everything.” (Interviewee 4)

It is felt that communication will be helped somewhat by involving the nurse or study coordinator, because they can play a very important role in promoting the quality of the information.

“usually we like that somebody […other] than the investigator or the physician ask for the consent […from] the patient. I think it’s the ideal solution when a research nurse or someone who is […] very aware of the project […and] know[s] the project very well, and then explains that they should take the time needed to answer the questions and so on, but I think […] that if not, the physician can also, the treating physician can ask for the consent. I don’t think it creates a problem.” (Interviewee 6)

An important barrier to this recruitment process, however, are time constraints faced by clinicians who are balancing clinical and research roles in a busy clinical environment. Clinicians generally do not have the time to fully explain a project and provide information about the research.

“They’re simply very busy and just don’t have time to think about it.” (Interviewee 7)

Finally, lack of awareness on the part of the clinicians about the research project can lead to the obstruction of research.

“My experience is that the greatest obstruction to clinical research is not the patient, it is the other physician…you have to get other physicians on board” (Interviewee 7)

To foster commitment among clinicians, there is need to integrate research and clinical work. Within such a model, a mission statement (patient-centered care generating new knowledge) and standard operating procedures should be developed for clinical research and revised appropriately. In order to help with recruitment, it is useful to build physician awareness and enthusiasm about the research, and to encourage their voluntary participation in it.

“it’s really important that [physicians] want to get involved in the study because it will require time, it will require energy, so that’s why it’s very important that it’s on a voluntary basis.” (Interviewee 2)

### Conflict of interest

Some respondents mentioned that conflicts of interest may exist specifically at the time the clinicians recruit patients to the research project because they have a therapeutic relationship. Several respondents discussed ways to address this concern. One suggested that it is essential for physicians to go through the Informed Consent Form with patients, highlighting that there will be no direct therapeutic benefit for the patient, and this may influence the patient’s decision to participate or not.

“We clearly say in [the] consent forms and verbally, [that] we do not expect any clinical benefit […for] you. Patients still hold that hope. It’s human nature.” (Interviewee 1)

Another respondent suggested ways to address this issue. One approach that was recommended is for the clinician-researcher to avoid direct recruiting contact. Another approach is for a neutral party to seek the patient’s consent. This position promotes the research nurse who can make an important contribution and help address the conflicts of interest because they do not use this information for their own gain. Research nurses are well positioned to obtain the patient’s consent because they are aware and well-informed about the project, and can provide the patient with answers to their questions and the time needed to deliberate participation.

“Usually we like that somebody else [other] than the investigator or the physician ask for the consent […from] the patient.” (Interviewee 6)

Although research nurses play a very important role, it was also acknowledged that if the treating physician does not have access to a research nurse, he or she can also be an option for obtaining the consent.

### Education and training

The importance of education and training was mentioned several times. It was stated that there appears to be a lack of physicians with training in research and research ethics. It was felt that training should occur within the medical school program and more should be done to increase the number of physicians with such training. Also, it was recognized that one important barrier is that physicians typically appear to favour working with patients in a clinical or hospital setting over getting involved in research.

“Most physicians, when they choose […] medicine as a profession, they choose medicine to see patients, to provide care, [and] not to do research. So it’s really a minority of individuals who are really interested in […] research.” (Interviewee 6)

In addition to these skills, training should be complimented by ongoing reminders about current research projects and provided to physicians, who are normally busy with their own practices and might not keep track of research opportunities for their patients. Furthermore, it is important for researchers and physicians to know what they are looking for in the study so that they can effectively answer the patient’s questions.

### Research ethics boards (REB)

There were comments about the variability in how REBs assess non-therapeutic research with some REBs more open to the idea of such research.

“There are interests for […] having non-therapeutical protocols out there. I guess some [REBs] might be more open to the option than others.” (Interviewee 4)

Several respondents indicated that research ethics boards are necessary for the general oversight of non-therapeutic research.

“if there is an increased risk, I think that usually the Ethic[s] Committee has to evaluate whether the risk is too important and usually you would not be allowed to start a project that has too high a risk for the patients” (Interviewee 6)

### Funds

This type of recruitment is time consuming and requires a team (e.g. physicians, researchers, research nurse) and resources (e.g. work space) to enable a smooth and successful process. For this reason, budgets are an issue.

“sometimes also you need more people to work but you don’t have space. And the budget is not good enough.” (Interviewee 3)

## Discussion

Although international guidelines address the recruitment of patients into non-therapeutic research, difficulties remain in applying them and ethical issues are still at play [20]. Our findings suggest that challenges in conducting non-therapeutic research are distinct from those derived from research for therapeutic reasons and reveal a number of ethical and practical challenges for physicians and researchers alike. We characterized these challenges as barriers and facilitators surrounding ethical considerations, patient-centered care, and the professional relationship.

Our analysis identified that respondents seemed well informed of the ethical principles of autonomy, beneficence, the need to respect the patient and to put his or her interests ahead of those of society. Risk of discrimination by employers and insurance companies was expressed as well as concerns about the confidentiality of the research results, and the impact of such information on patients’ families. These findings are supported by the literature surrounding issues of confidentiality and discrimination [7,8]. Furthermore, due to the close ties between ethics and clinical practice, physicians should only partake in recruitment on a voluntary basis; otherwise they may inadvertently convey their disinterest to the potential participants, and negatively impact participation. Finally, there were a number of respondents who indicated that giving back to society and the desire to help future generations, even if there are no specific benefits for the patients themselves, were important moral values for patients who affirmed their willingness to participate.

In addition to ethical challenges, there were specific facilitators and barriers for recruiting patients to non-therapeutic research. Facilitators were most often associated with patient-centered issues enhancing communication, while barriers to non-therapeutic research were most often professionally based.

Families were identified as a positive factor and a source of information throughout the process. The family unit was perceived as an essential source of support for the patient, along with real feedback and true stories from individuals who have already gone through similar experiences. They provide the patient with a sense of reassurance, thus fostering integration and improving participation in such studies.

Another facilitator for recruitment is the role played by research nurses. Notably, they are seen as a source of support or as an “extra pair of hands” involved in the care of the patient. Respondents identified them as a more neutral party than researchers, having less of a vested interest in the research, and making them an ideal candidate for obtaining participants’ consent. Nonetheless, it is important to note that nurses, like physician-researchers, may be susceptible to conflicts of interest and steps should be taken to ensure that any such conflict is limited and disclosed to potential participants [23]. Respondents also supported the idea that the informed consent process should be active, voluntary and that it is important to take the time to answer questions, transmit the information, and be available for the patient. These findings affirm the issues of time constraints and information transmission explored in the literature [7].

The team approach can enhance communication to patients. The image of a team, working together throughout every step, is favoured for non-therapeutic research. Respondents indicated that this communication should be transparent among all parties, resulting in patients who are well informed and supported. These findings resonate with the literature on doctor-patient communication [24]. Another facilitator is the importance of knowledge translation and educating the public about the research. Greater awareness among the patients of the different types of research going on could help to reduce any negative ideas and misconceptions that may exist (i.e. the guinea pig perception) and be an effective approach to improving recruitment.

Our findings also indicate that respondents acknowledge the importance of the doctor-patient relationship in promoting trust [25]. However, there are several challenges associated with integrating the clinician/researcher role into research. Respondents suggested that a therapeutic misconception may occur because the trust the patient has toward the physician can have an influence. If the physician suggests it, then it must be the right option. This challenge, for example, stems from the need to take the time to fully discuss the research and the recognised lack of time to do so, due to busy schedules. In order to promote participation in research, physicians (families, nurses, and REBs too) should try not to intervene and influence the patient’s decision.

Furthermore, the lack of awareness of research by other physicians was seen as the “greatest obstruction” to research recruitment. A solution proposed for remedying this barrier is to try to get physicians on board with the research that is taking place. Such a strategy may help enhance recruitment and the advancement of research.

Moreover, there appears to be a generation gap among physicians, with the younger ones having less of an interest in and awareness of research; therefore, an effort should be made to reverse this trend and further educate physicians. Respondents noted that it is useful to foster a commitment to training programs that expose medical students to research and encourage an interest in it. The current study sheds light on the importance of research ethics in the medical curriculum. Although the ideal time to introduce research ethics into medical training can be a source of debate, it is generally introduced during the undergraduate curriculum and then further reinforced during residency, fellowships, and specialty training [26]. This training is also encouraged to be a continuous process and the teaching to promote active participation in the learning [26]. Finally, despite the undergraduate medical curricula workload, integrating research ethics can be seen as necessary in order to train physicians who are more sensitive to and conscious of the ethical aspects that exist in their daily practice [26].

A final barrier to recruitment is the need to address the vulnerability of the patient and to be sensitive to a variety of emotional responses throughout the recruitment process such as depression, distress, and reassurance. Existing research supports our finding that patients experience a range of emotional readiness [27]. Given the important role of families, it will be important to incorporate them in the process as much as possible [28].

Based on our findings, we offer several points to consider in order to overcome barriers to recruitment for non-therapeutic research (see Figure 1). These examples are divided into patient-centered issues and health professional issues influencing non-therapeutic research. Each of the divisions outlines the issues at hand and provides strategies for dealing with and achieving a better understanding of the challenges. Promoting communication and autonomy and fostering familial support are important elements for overcoming patient-centered issues, while facilitating the doctor-patient relationship and teamwork and implementing educational programs are seen as key factors for dealing with health professional issues.

This study has limitations. First, it is an exploratory study focused on a small research community. It was not large enough to allow saturation of subtle differences between sub-groups (e.g. oncologists, geneticists, clinicians not involved in research). For this reason, the results do not represent the views of a more general community of researchers. Also, the use of volunteer health professional respondents may have resulted in an overrepresentation of those involved in clinical research. Further research with clinicians who are not actually involved in research is needed, to assess their views on the recruitment of their patients for non-therapeutic research. Finally, it is possible that additional interviews with researchers and/or clinicians could generate other themes that were not identified in this project. Nevertheless, this study fills an important gap in the literature by addressing the barriers and facilitators to non-therapeutic research under three prominent themes: ethical considerations, patient-centered issues, and health professional issues.

## Conclusions

In conclusion, while patients are truly vulnerable, we take the view that non-therapeutic research is ethically justified primarily because of the benefit for society. If we did not ask seriously ill patients to participate, then medical science could not advance, at least in those fields where the only possible avenue for research (at least at this time) requires the use of diseased tissue. This is not to say that just any research can proceed when the harms outweigh the benefits; the risks must be justifiable. Therefore, the protection of research participants from excessive harms is very important. This is a responsibility of both the researchers and the REBs that oversee the research (including protocols and informed consent forms). Based on anecdotal evidence from our research team, it appears that for this research some REBs are hesitant to approve a study where there is greater than minimal risk with no direct benefit; however, the autonomy of the patient/participant combined with mechanisms to ensure that they are appropriately informed of the risks can ensure that participation is fully voluntary and informed. Furthermore, this type of research is supported in Canada by article 11.4 of the *Tri-Council Policy Statement: Ethical Conduct for Research Involving Humans* (TCPS 2), which sees it as justified when researchers and REBs ensure that the potential risk is appropriately minimized and is outweighed by the potential benefits. The TCPS 2 goes on to state that ethical principles of respect for persons and concern for welfare guide this research, as well as any research with direct benefit [29]. Finally, it has recently been advocated by ethics commentators that an obligation to participate in biomedical research should exist because research produces a public good that is accessible to everyone [30].

This exploratory study reveals the challenges of wrestling with the recruitment of patients to non-therapeutic research. As the science continues to evolve, closer collaboration with clinicians will be required in order to ensure respect of patients and more social and emotional support for them throughout the research process and the course of their disease.

Due to the important frontline role that researcher-clinicians play, it is imperative to introduce: a better understanding of the challenges researchers/clinicians face when recruiting patients for non-therapeutic research; help improve effective communication among all members of the team, as well as with patients and their families (effective communication training programs/workshops); and facilitate better access to training programs about the value of non-therapeutic research. An important next step is to initiate research exploring the views of participants regarding non-therapeutic research, as well as the views of clinicians not involved in non-therapeutic research.

## Competing interests

The authors declare that they have no competing interests.

## Authors’ contributions

EK participated in the development of a conceptual framework, the analysis of data, and the drafting of the manuscript. DA conceived the study, participated in the design of the study, the development of a conceptual framework, the collection and analysis of data, and the drafting of the manuscript. LB was involved in the design of the study, the collection of data, and the revision of the manuscript. ZD and CR participated in the coordination of the study and provided support and critical analysis of the manuscript. BMK provided support and critical analysis of the manuscript. EK wrote the first draft of the manuscript; DA and LB commented on the drafts. All authors read and approved the final manuscript.

## Pre-publication history

The pre-publication history for this paper can be accessed here:

http://www.biomedcentral.com/1472-6939/13/33/prepub
